# Supplementary material for: Phylogeny, Taxonomy and Morphological Characteristics of Apiospora (Amphisphaeriales, Apiosporaceae)
Source: Microorganisms. 2024 Jul 4;12(7):1372. doi: 10.3390/microorganisms12071372 (PMC11278877; doi:10.3390/microorganisms12071372)
Supplement: Supplementary file 1 [file microorganisms-12-01372-s001.zip › Table S1.docx]

**Table S1.** Species and GenBank accession numbers of DNA sequences used in this study.

| Species | Strain no. | Substrate | Country | GenBank accession numbers | | | |
| --- | --- | --- | --- | --- | --- | --- | --- |
|  |  |  |  | ITS | LSU | TEF1α | TUB2 |
| *Apiospora acutiapica* | KUMCC 20-0210 * | *Bambusa bambos* | China | MT946343 | MT946339 | MT947360 | MT947366 |
| *A. adinandrae* | SAUCC 1282B-1 * | *Adinandra glischroloma* | China | OR739431 | OR739572 | OR753448 | OR757128 |
|  | SAUCC 1282B-2 | *Adinandra glischroloma* | China | OR739432 | OR739573 | OR753449 | OR757129 |
| *A. agari* | KUC21333 * | *Agarum cribrosum* | Korea | MH498520 | MH498440 | MH544663 | MH498478 |
| *A. ananasi* | MFLU 23-0236* |  |  | OR438409 | OR438876 | OR500338 | OR538086 |
|  | MFLUCC 23-0101 |  |  | OR438410 | OR438877 | OR500339 | OR538085 |
| *A. aquatica* | S-642 * | Submerged wood | China | MK828608 | MK835806 | – | – |
| *A. arctoscopi* | KUC21331 * | Egg of *Arctoscopus japonicus* | Korea | MH498529 | MH498449 | MN868918 | MH498487 |
| ***A. armeniaca*** | **SAUCC DL1831*** | ***Prunus armeniaca*** | **China** | **OQ592540** | **OQ615269** | **OQ613313** | **OQ613285** |
|  | **SAUCC DL1844** | ***Prunus armeniaca*** | **China** | **OQ592539** | **OQ615268** | **OQ613312** | **OQ613284** |
| *A. arundinis* | CBS 124788 | *Fagus sylvatica* | Switzerland | KF144885 | KF144929 | KF145017 | KF144975 |
| *A. aurea* | CBS 244.83 * | Air | Spain | AB220251 | KF144935 | KF145023 | KF144981 |
| ***A. babylonica*** | **SAUCC DL1841*** | ***Salix babylonica*** | **China** | **OQ592538** | **OQ615267** | **OQ613311** | **OQ613283** |
|  | **SAUCC DL1864** | **saprophytic leaves** | **China** | **OQ592537** | **OQ615266** | **OQ613310** | **OQ613282** |
| *A. balearica* | CBS 145129 * | Undetermined *Poaceae* | Spain | MK014869 | MK014836 | MK017946 | MK017975 |
| *A. bambusicola* | MFLUCC20-0144 * | *Schizostachyum brachycladum* | Thailand | MW173030 | MW173087 | MW183262 | – |
| *A. bawanglingensis* | SAUCC BW0444 * | *Indocalamus longiauritus* | China | OR739429 | OR739570 | OR753446 | OR757126 |
|  | SAUCC BW04441 | *Indocalamus longiauritus* | China | OQ592551 | OQ615280 | OQ613324 | OQ613302 |
| *A. biserialis* | CGMCC 3.20135 * | Bamboo | China | MW481708 | MW478885 | MW522938 | MW522955 |
| *A. camelliae-sinensis* | LC5007 * | *Camellia sinensis* | China | KY494704 | KY494780 | KY705103 | KY705173 |
| *A. chiangraiense* | MFLUCC21-0053 * | Dead culms of bamboo | Thailand | MZ542520 | MZ542524 | – | MZ546409 |
| *A. chromolaenae* | MFLUCC 17-1505* | *Chromolaena odorata* | Thailand | MT214342 | MT214436 | – | – |
| *A. cordylines* | GUCC 10027 * | *Cordyline fruticosa* | China | MT040106 | – | MT040127 | MT040148 |
| *A. cyclobalanopsidis* | CGMCC 3.20136 * | *Cyclobalanopsidis glauca* | China | MW481713 | MW478892 | MW522945 | MW522962 |
| *A. descalsii* | CBS 145130 * | *Ampelodesmos mauritanicus* | Spain | MK014870 | MK014837 | MK017947 | MK017976 |
| *A. dichotomanthi* | LC4950 * | *Dichotomanthus tristaniaecarpa* | China | KY494697 | KY494773 | KY705096 | KY705167 |
| *A. dongyingensis* | SAUCC 0302 * | Leaf of bamboo | China | OP563375 | OP572424 | OP573264 | OP573270 |
|  | SAUCC 0303 | Leaf of bamboo | China | OP563374 | OP572423 | OP573263 | OP573269 |
| *A. esporlensis* | CBS 145136 * | *Phyllostachys aurea* | Spain | MK014878 | MK014845 | MK017954 | MK017983 |
| *A. euphorbiae* | IMI 285638b | *Bambusa* sp. | Bangladesh | AB220241 | AB220335 | – | AB220288 |
| *A. fermenti* | KUC21289 * | Seaweed | Korea | MF615226 | MF615213 | MH544667 | MF615231 |
| *A. gaoyouensis* | CFCC 52301 * | *Phragmites australis* | China | MH197124 | – | MH236793 | MH236789 |
| *A. garethjonesii* | JHB004 * | Culms of dead bamboo | China | KY356086 | KY356091 | – | – |
| *A. gelatinosa* | HKAS 111962 * | Culms of dead bamboo | China | MW481706 | MW478888 | MW522941 | MW522958 |
| *A. guiyangensis* | HKAS 102403 * | Dead culms of *Poaceae* | China | MW240647 | MW240577 | MW759535 | MW775604 |
|  | LC5322 * | Air in karst cave | China | KY494709 | KY494785 | KY705108 | KY705178 |
| *A. hainanensis* | SAUCC 1681 * | Leaf of bamboo | China | OP563373 | OP572422 | OP573262 | OP573268 |
|  | SAUCC 1682 | Leaf of bamboo | China | OP563372 | OP572421 | OP573261 | OP573267 |
| *A. hispanica* | IMI 326877 * | Maritime sand | Spain | AB220242 | AB220336 | – | AB220289 |
| *A. hydei* | CBS 114990 * | Culms of *Bambusa tuldoides* | China | KF144890 | KF144936 | KF145024 | KF144982 |
| *A. hyphopodia* | MFLUCC 15-0003* | Dead culms of bamboo | Thailand | KR069110 | – | – | – |
| *A. hysterina* | ICPM 6889 * | Bamboo | New Zealand | MK014874 | MK014841 | MK017951 | MK017980 |
| *A. iberica* | AP10118 * | *Arundo donax* | Portugal | MK014879 | MK014846 | MK017955 | MK017984 |
| *A. indocalami* | SAUCC BW0455 * | *Indocalamus longiauritus* | China | OR739430 | OR739571 | OR753447 | OR757127 |
|  | SAUCC BW04551 | *Indocalamus longiauritus* | China | OQ592550 | OQ615279 | OQ613323 | OQ613301 |
| *A. intestine* | CBS 135835 * | Gut of grasshopper | India | KR011352 | KR149063 | KR011351 | KR011350 |
| *A. italica* | CBS 145138 * | *Arundo donax* | Italy | MK014880 | MK014847 | MK017956 | MK017985 |
| *A. jatrophae* | CBS 134262 * | *Jatropha podagrica* | India | JQ246355 | – | – | – |
| *A. jiangxiensis* | LC4577 * | *Maesa* sp. | China | KY494693 | KY494769 | KY705092 | KY705163 |
| ***A. jinanensis*** | **SAUCC DL1981*** | ***Bambusaceae* sp.** | **China** | **OQ592544** | **OQ615273** | **OQ613317** | **OQ613289** |
|  | **SAUCC DL2000** | ***Bambusaceae* sp.** | **China** | **OQ592543** | **OQ615272** | **OQ613316** | **OQ613288** |
| *A. kogelbergensis* | CBS 113333 * | Dead culms of *Restionaceae* | South Africa | KF144892 | KF144938 | KF145026 | KF144984 |
| *A. koreana* | KUC21332 * | Egg of *Arctoscopus japonicus* | Korea | MH498524 | MH498444 | MH544664 | MH498482 |
| *A. locuta-pollinis* | LC11683 * | *Brassica campestris* | China | MF939595 | – | MF939616 | MF939622 |
| *A. longistroma* | MFLUCC 11-0481* | Culms of decaying bamboo | Thailand | KU940141 | KU863129 | – | – |
| *A. malaysiana* | CBS 102053 * | *Macaranga hullettii* | Malaysia | KF144896 | KF144942 | KF145030 | KF144988 |
| *A. machili* | SAUCC 1175A-4* | *Machilus nanmu* | China | OR739433 | OR739574 | OR753450 | OR757130 |
|  | SAUCC 1175 | *Machilus nanmu* | China | OQ592560 | OQ615289 | OQ613333 | OQ613307 |
| *A. marianiae* | AP18219 * | Dead stems of *Phleum pratense* | Spain | ON692406 | ON692422 | ON677180 | ON677186 |
| *A. marii* | CBS 497.90 * | beach sands | Spain | MH873913 | KF144947 | KF145035 | KF144993 |
| *A. marina* | KUC21328 * | Seaweed | Korea | MH498538 | MH498458 | MH544669 | MH498496 |
| *A. mediterranea* | IMI 326875 * | Air | Spain | AB220243 | AB220337 | – | AB220290 |
| *A. minutispora* | 17E-042 * | Soil | South Korea | LC517882 | – | LC518889 | LC518888 |
| *A. montagnei* | AP301120 * | *Arundo micrantha* | Spain | ON692408 | ON692424 | ON677182 | ON677188 |
|  | AP19421 | *Arundo micrantha* | Spain | ON692418 | ON692425 | ON677183 | ON677189 |
|  | CPC 18900 | Culms of *Phragmites australis* | Italy | KF144909 | KF144956 | KF145043 | KF145001 |
| *A. mori* | MFLU 18-2514 * | Dead leaves of *Morus australis* | China | MW114313 | MW114393 | – | – |
| *A. multiloculate* | MFLUCC 21-0023* | Dead culms of Bambusae | Thailand | OL873137 | OL873138 | – | OL874718 |
| *A. mytilomorpha* | DAOM 214595 * | Dead blades of *Andropogon* sp. | India | KY494685 | – | – | – |
| *A. neobambusae* | LC7106 * | Leaf of bamboo | China | KY494718 | KY494794 | KY806204 | KY705186 |
| *A. neochinense* | CFCC 53036 * | *Fargesia qinlingensis* | China | MK819291 | – | MK818545 | MK818547 |
| *A. neogarethjonesii* | HKAS 102408 * | Dead culms of Bambusae | China | MK070897 | MK070898 | – | – |
| *A. neosubglobosa* | KUMCC 16-0203 * | Bamboo | China | KY356090 | KY356095 | – | – |
| *A. obovate* | LC4940 * | *Lithocarpus* sp. | China | KY494696 | KY494772 | KY705095 | KY705166 |
| *A. ovata* | CBS 115042 * | *Arundinaria hindsii* | China | KF144903 | KF144950 | KF145037 | KF144995 |
| *A. paraphaeosperma* | MFLUCC13-0644 * | Dead clumps of *Bambusa* sp. | Thailand | KX822128 | KX822124 | – | – |
| *A. phyllostachydis* | MFLUCC 18-1101* | *Phyllostachys heteroclada* | China | MK351842 | MH368077 | MK340918 | MK291949 |
| *A. piptatheri* | CBS 145149 * | *Piptatherum miliaceum* | Spain | MK014893 | MK014860 | MK017969 | – |
| *A. pseudomarii* | GUCC 10228 * | Leaves of *Aristolochia debilis* | China | MT040124 | – | MT040145 | MT040166 |
| *A. pseudoparenchymatica* | LC7234 * | Leaf of bamboo | China | KY494743 | KY494819 | KY705139 | KY705211 |
| *A. pseudorasikravindrae* | KUMCC 20-0208 * | *Bambusa dolichoclada* | China | MT946344 | – | MT947361 | MT947367 |
| *A. pseudosinensis* | CPC 21546 * | Leaf of bamboo | Netherlands | KF144910 | KF144957 | KF145044 | MN868936 |
| *A. pseudospegazzinii* | CBS 102052 * | *Macaranga hullettii* | Malaysia | KF144911 | KF144958 | KF145045 | KF145002 |
| *A. pterosperma* | CPC 20193 * | Lepidosperma gladiatum | Australia | KF144913 | KF144960 | KF145046 | KF145004 |
| *A. pusillisperma* | KUC21321 * | Seaweed | Korea | MH498533 | MH498453 | MN868930 | MH498491 |
| *A. qinlingensis* | CFCC 52303 * | Fargesia qinlingensis | China | MH197120 | – | MH236795 | MH236791 |
| *A. rasikravindrae* | LC5449 | Soil in karst cave | China | KY494713 | KY494789 | KY705112 | KY705182 |
| *A. sacchari* | CBS 212.30 | Phragmites australis | UK | KF144916 | KF144962 | KF145047 | KF145005 |
| *A. saccharicola* | CBS191.73 | Air | Netherlands | KF144920 | KF144966 | KF145051 | KF145009 |
| *A. sargassi* | KUC21228 * | *Sargassum fulvellum* | Korea | KT207746 | KT207696 | MH544677 | KT207644 |
| *A. sasae* | CBS 146808 * | Dead culms of *Sasa veitchii* | Netherlands | MW883402 | MW883797 | MW890104 | MW890120 |
| *A. septate* | CGMCC 3.20134 * | Bamboo | China | MW481711 | MW478890 | MW522943 | MW522960 |
| *A. serenensis* | IMI 326869 * | Food, pharmaceutical excipients, atmosphere and home dust | Spain | AB220250 | AB220344 | – | AB220297 |
| *A. setariae* | CFCC 54041 * | Decaying culms of *Setaria viridis* | China | MT492004 | – | – | – |
| *A.setostroma* | KUMCC 19-0217 | Dead branches of bamboo | China | MN528012 | MN528011 | MN527357 | – |
| *A. sichuanensis* | HKAS 107008 * | Dead culms of *Poaceae* | China | MW240648 | MW240578 | MW759536 | MW775605 |
| *A. sorghi* | URM 93000 * | *Sorghum bicolor* | Brazil | MK371706 | – | – | MK348526 |
| *A. sphaerosperma* | CBS114314 | Leaf of *Hordeum vulgare* | Iran | KF144904 | KF144951 | KF145038 | KF144996 |
| *A. stipae* | CBS 146804 * | Dead culm of *Stipa gigantea* | Spain | MW883403 | MW883798 | MW890082 | MW890121 |
| *A. subglobosa* | MFLUCC11-0397 | Dead culms of bamboo | Thailand | KR069112 | KR069113 | – | – |
| *A. subrosea* | LC7292 * | Leaf of bamboo | China | KY494752 | KY494828 | KY705148 | KY705220 |
| *A. taeanense* | KUC 21322 * | Seaweed | South Korea | MH498515 | – | MH544662 | MH498473 |
| *A. thailandica* | LC5630 | Rotten wood | China | KY494714 | KY494790 | KY705113 | KY806200 |
| *A. vietnamensis* | IMI 99670 * | *Citrus sinensis* | Vietnam | KX986096 | KX986111 | – | KY019466 |
| *A. xenocordella* | CBS 478.86 * | Soil from roadway | Zimbabwe | KF144925 | KF144970 | KF145055 | KF145013 |
| *A. yunnana* | MFLUCC 15-0002* | Decaying bamboo culms | China | KU940147 | KU863135 | – | – |
| *Arthrinium caricicola* | CBS 145127 | *Carex ericetorum* | China | MK014871 | MK014838 | MK017948 | MK017977 |

Notes: Ex‐type strains are marked with “*”, strains in this study are marked in bold and “–” .
