# Supplementary figures and images for: Phylogeny, Taxonomy and Morphological Characteristics of Apiospora (Amphisphaeriales, Apiosporaceae)
Source: Microorganisms. 2024 Jul 4;12(7):1372. doi: 10.3390/microorganisms12071372 (PMC11278877; doi:10.3390/microorganisms12071372)

Tree scale: 0.1

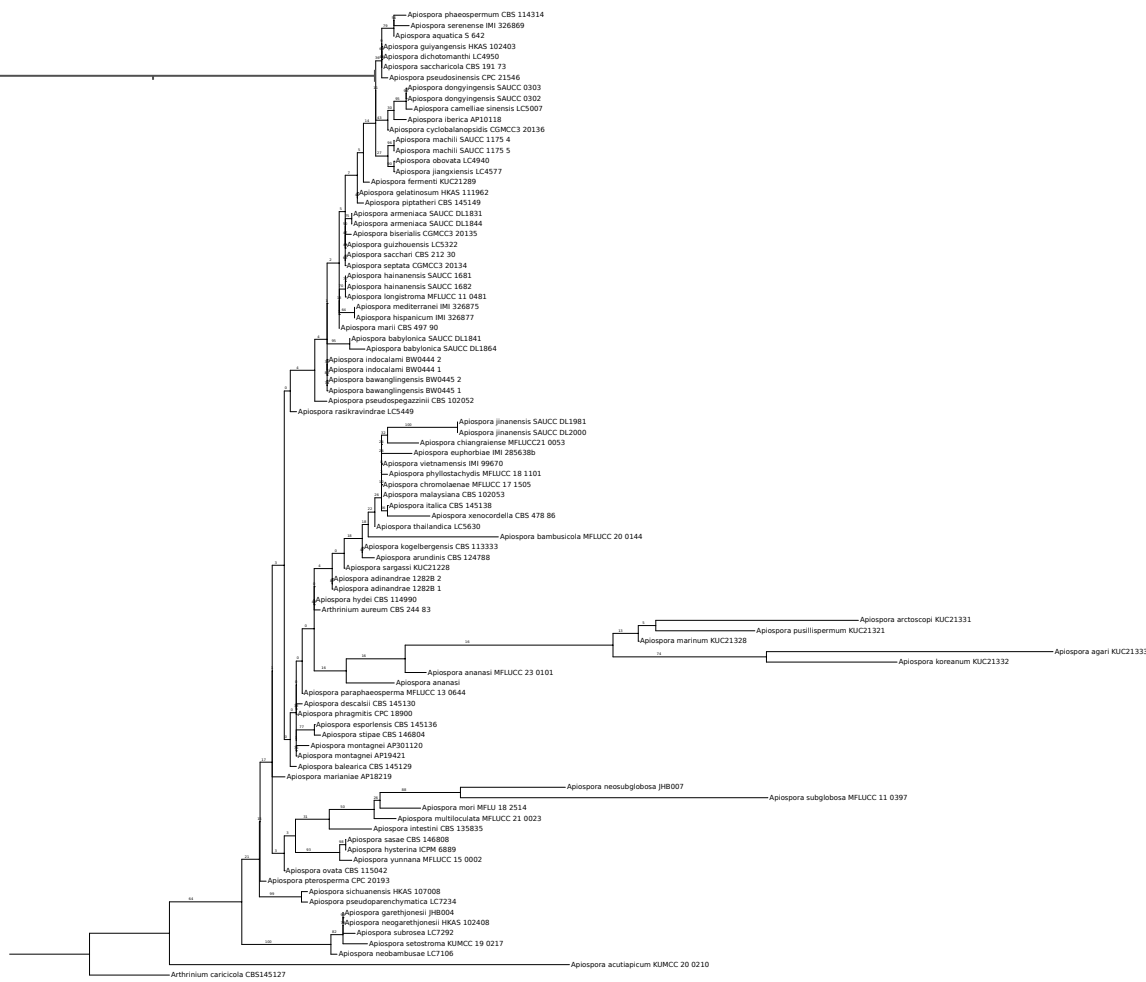

Supplement: Supplementary file 1 [file microorganisms-12-01372-s001.zip › Figure S2.The ML tree of LSU sequence.pdf]

Tree scale: 1

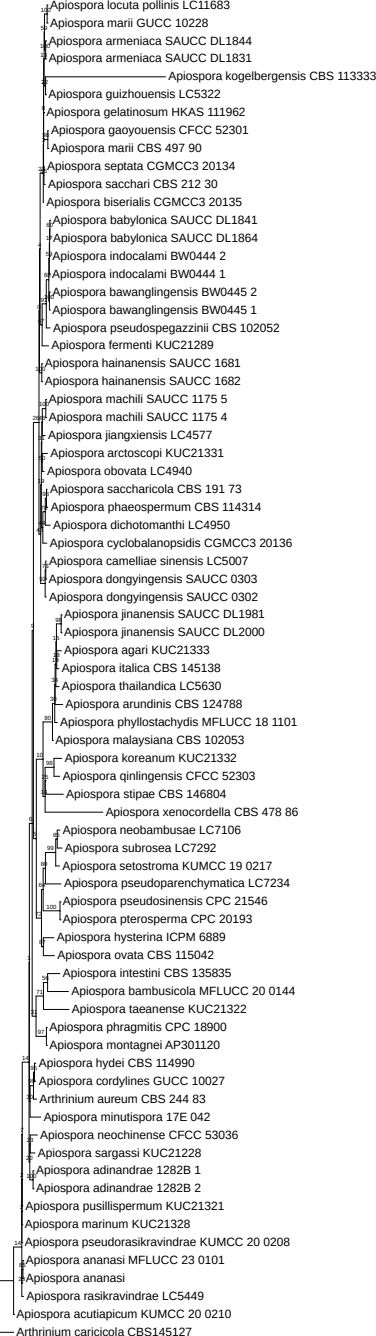

Supplement: Supplementary file 1 [file microorganisms-12-01372-s001.zip › Figure S3. The ML tree of tef sequence.pdf]

Tree scale: 0.1

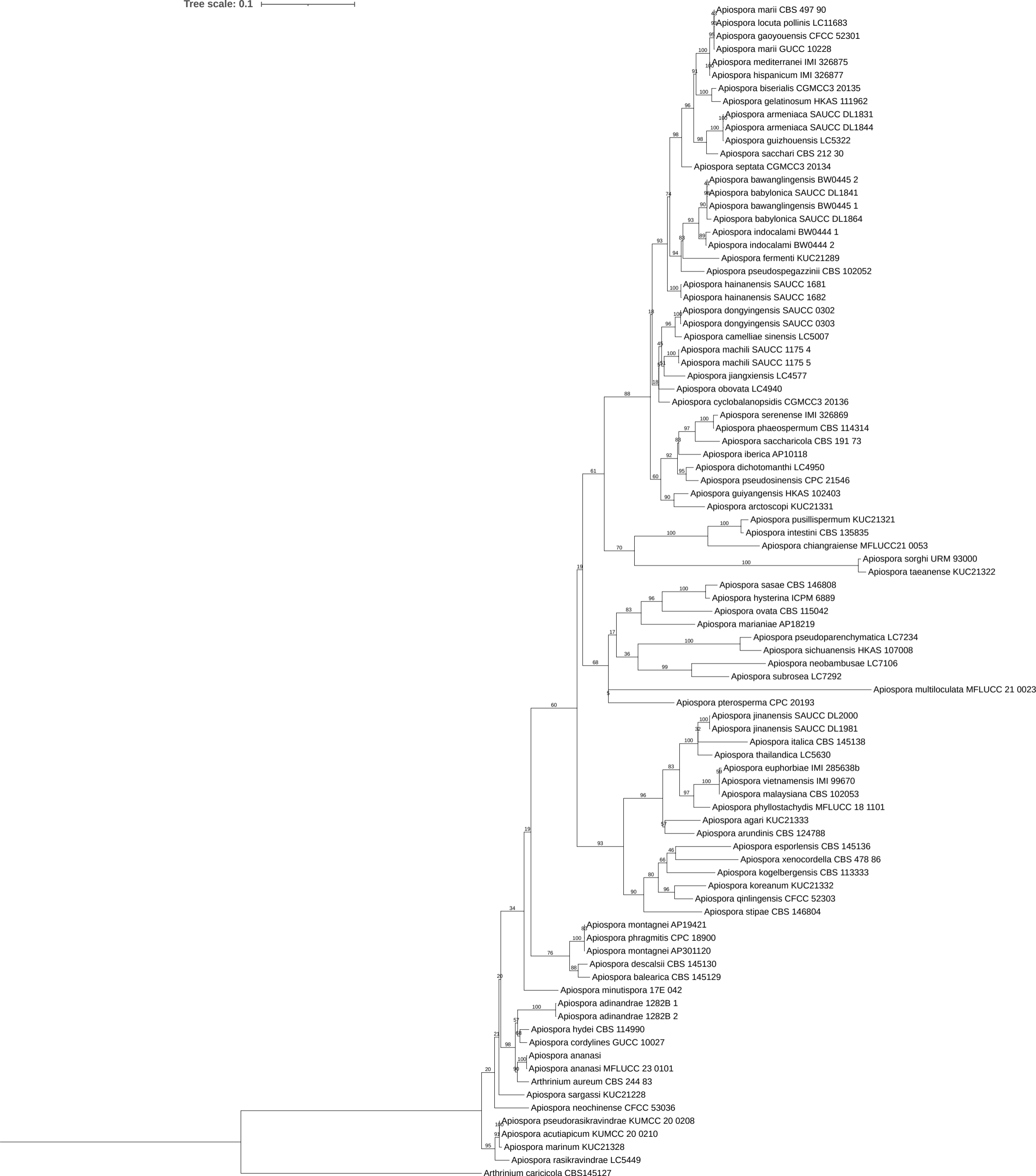

Supplement: Supplementary file 1 [file microorganisms-12-01372-s001.zip › Figure S4. The ML tree of tub sequence.pdf]
